# Five-Fraction Radiosurgery Using a Biologically Equivalent Dose of a Single Fraction of 24 Gy for a 3-cm Parasagittal Para-Central Sulcus Brain Metastasis From Adenocarcinoma of the Cecum

**DOI:** 10.7759/cureus.48799

**Published:** 2023-11-14

**Authors:** Kazuhiro Ohtakara, Takanori Kondo, Yuma Obata, Kentaro Fujii, Kojiro Suzuki

**Affiliations:** 1 Department of Radiation Oncology, Kainan Hospital Aichi Prefectural Welfare Federation of Agricultural Cooperatives, Yatomi, JPN; 2 Department of Radiology, Aichi Medical University, Nagakute, JPN; 3 Department of Surgery, Kainan Hospital Aichi Prefectural Welfare Federation of Agricultural Cooperatives, Yatomi, JPN; 4 Department of Surgery, Gifu Prefectural Tajimi Hospital, Tajimi, JPN; 5 Department of Neurosurgery, Kainan Hospital Aichi Prefectural Welfare Federation of Agricultural Cooperatives, Yatomi, JPN

**Keywords:** brain radionecrosis, colorectal cancer, eloquent cortex, biologically effective dose, stereotactic radiosurgery, hypofractionation, cecal cancer, brain metastases, bevacizumab, adverse radiation effect

## Abstract

An isolated single brain metastasis (BM) is an extremely rare manifestation of failure in patients with cecal adenocarcinoma (CAC). Total *en bloc* resection (while preserving function) of a 3-cm BM involving both the primary motor and sensory cortexes presents a conundrum: achieving long-term local control and safety of such a BM is also challenging for stereotactic radiosurgery (SRS). We describe the case of a 3.1-cm BM from CAC in the left parasagittal para-central sulcus region, which was treated using five-fraction SRS with a biologically effective dose (BED) of 81.6 Gy. In the SRS, the gross tumor volume (GTV, 7.14 cm^3^) was defined based on computed tomography (CT)/T1/T2 matching (enhancing lesion 11.66 cm^3^), and 98.7% of the GTV (CT/T2 mass) was covered with 43.6 Gy (58% isodose) using volumetric-modulated arcs. The maximum tumor response was partial (19.7% of the prior GTV) and sustained for 15.2 months, leaving minor neurological symptoms. However, the patient developed neurological worsening at six months, attributed to adverse radiation effects with a CT/T1/T2 mismatch, for which medical management, including the addition of bevacizumab (BEV), was effective for one year. Multi-fraction SRS with a high marginal and internal BED and sequential systemic therapy, including BEV, can be a minimally invasive, efficacious, and durable treatment option for a large CAC-BM involving the central sulcus. Early co-administration of BEV following SRS, dose escalation to the GTV boundary, and more than five fractions of SRS may be considered to improve the efficacy and safety further.

## Introduction

Brain metastases (BMs) are a rare, hematogenous spread from colorectal cancer (CRC), especially when developing without liver or lung metastases [1]. However, the incidence of BMs from CRC is anticipated to occur, owing to improved survival of patients with locally advanced or metastatic CRC, driven by ongoing advancement of systemic therapy [1]. Even in recent reports, the prognosis of patients harboring CRC BMs generally remains dismal, with a median survival of 5-10 months [2-6]. In addition, right-sided CRC (including the appendix, cecum, and ascending colon) is associated with an unfavorable prognosis, compared with left-sided CRC (including the descending colon, sigmoid, and rectum) [7]. BMs from cecal cancer without any other simultaneous, distant metastases are extremely rare in either a synchronous or metachronous setting [8,9].

Given the limited survival of patients with CRC BMs, developing efficacious, safe, and minimally invasive treatments for CRC BMs is an important issue. Clinical management of patients harboring a symptomatic CRC BM >2 cm in maximum diameter, localized in the parasagittal para-central sulcus region, is a challenge. Although total *en bloc* resection (while preserving function) of the BM is preferable for immediate decompression and symptom improvement, a superior cerebral vein crossing directly above the lesion and draining into the superior sagittal sinus and/or poorly demarcated brain-tumor interface with profound microscopic brain invasion can compromise safe total resection [10]. Given the generally unfavorable radiosensitivity, achieving long-term local control and safety of such a BM from colorectal cancer is also challenging for stereotactic radiosurgery (SRS) [2,11].

We describe a case of a symptomatic 3.1-cm BM located in the parasagittal para-central sulcus region, developing metachronously from cecal adenocarcinoma without any other distant metastases, which was treated by five-fraction SRS with a biologically effective dose (BED) of 81.6 Gy to the gross tumor boundary. The BM showed a partial response, followed by symptomatic local progression six months after the SRS, for which medical management, including changes in chemotherapy, was effective. The gross tumor itself sustained regression during the 15.2-month follow-up period. We discuss ways to further improve the efficacy and safety of this treatment scheme.

This report was part of the clinical study approved by the Clinical Research Review Board of Kainan Hospital Aichi Prefectural Welfare Federation of Agricultural Cooperatives (20220727-1).

## Case presentation

**Figure 1:**
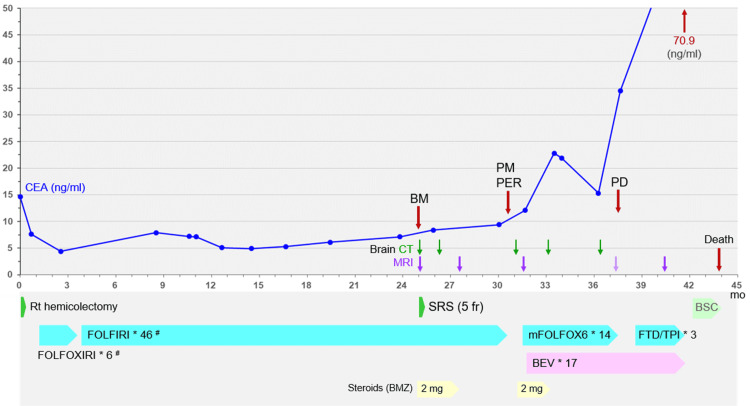
Summary of anti-cancer treatments along with a series of changes in the carcinoembryonic antigen level FOLFOXIRI: 5-fluorouracil (5-FU), calcium folinate/leucovorin (*l*-LV), oxaliplatin (L-OHP), and irinotecan (CPT-11); FOLFIRI: 5-FU, *l*-LV, and CPT-11; mFOLFOX6: modified FOLFOX (5-FU, *l*-LV, and L-OHP); FTD/TPI: trifluridine and tipiracil hydrochloride; BEV: bevacizumab (5 mg/kg) ^#^The dose of CPT-11 was reduced by 20%, due to a heterozygous carrier with the *6 allele variant of the *UGT1A1* gene. CEA: carcinoembryonic antigen; BM: brain metastasis; PM: pulmonary metastases; PER: peritoneal dissemination; PD: progressive disease; CT: computed tomography; MRI: magnetic resonance imaging; mo: months; Rt: right; SRS: stereotactic radiosurgery; fr: fraction; BSC: best supportive care; BMZ: betamethasone; *UGT1A1*, uridine disphosphate glucuronosyltransferase 1

A 68-year-old, right-handed male, with a Karnofsky performance status of 100%, underwent laparoscopic right hemicolectomy for cecal cancer that was detected by screening, which resulted in non-curative resection of the lymph node metastases and disseminated nodules involving the para-aortic major vessels, including ileocolic artery and superior mesenteric artery/vein. The pathological examination revealed moderately differentiated, mainly tubular and partly mucinous, adenocarcinoma, harboring Kirsten rat sarcoma viral oncogene (KRAS) G12D mutation while the other biomarkers, including a serine/threonine-protein kinase B-Raf (BRAF) mutation and microsatellite instability, were negative [1,4,5]. Due to the rapid progression of the unresectable lesions, the patient subsequently received adjuvant chemotherapy, in which bevacizumab (BEV) was excluded, considering the risk of hemorrhage from the vessel-involving lesions. The anti-cancer treatments are summarized in Figure 1.

**Figure 2:**
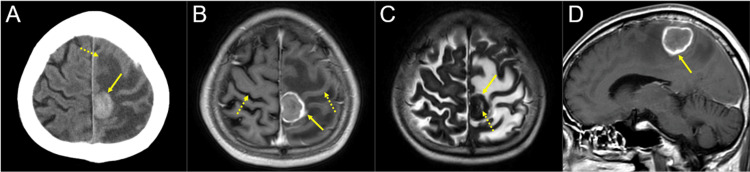
Computed tomography and magnetic resonance images at the diagnosis of the brain metastasis The images show a non-contrast-enhanced (CE) computed tomography (CT) image (A); CE-T1-weighted images (WIs) (B, D); a T2-WI (C); axial images (A-C); and a sagittal image (D). (A-D) These images are shown at the same magnification and coordinates under co-registration and fusions. (A) A high-density, well-demarcated solid mass lesion (arrow in A) located in the left parasagittal para-central sulcus region and associated with extensive perilesional edema (dashed arrow in A) in the ipsilateral fronto-parietal lobes (B, D). The mass itself is slightly enhanced, whereas the surrounding region is markedly enhanced with slight exudation of contrast media into the surrounding parenchyma (arrows in B, D). The bilateral central sulci are indicated by dashed arrows in B. (C) On T2-WIs, the mass (arrow in C) is generally low-intensity, containing the high-intensity region (dashed arrow in C), suggestive of central necrosis.

The chemotherapy resulted in complete remission (CR) of the residual lesions and was therefore continued. However, two years after the surgery, the patient noticed progressive motor weakness of the right lower extremity and was clinico-radiologically diagnosed with a single BM of 31 mm maximum diameter, located in the left parasagittal para-central sulcus region (mainly involving the precentral gyrus, paracentral lobule, precuneus, and postcentral gyrus) (Figure 2).

**Figure 3:**
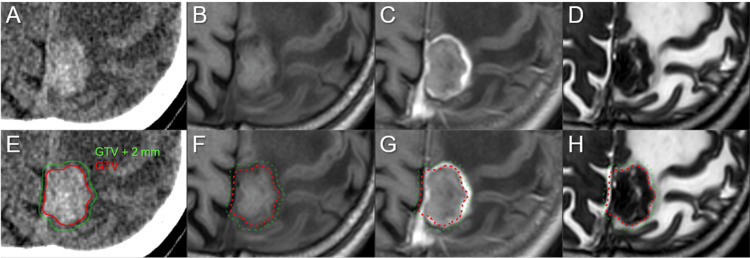
Target definition based on the multi-image co-registration, fusion, and comparison for stereotactic radiosurgery The axial images show non-CE-CT images (A, E); non-CE-T1-WIs (B, F); CE-T1-WIs (C, G); T2-WIs (D, H); and target contours superimposed onto the images (E-H). (A-H) These images are shown at the same magnification and coordinates under co-registration and fusions. The gross tumor volume (GTV) is defined based on a comparison of the visible masses on CT images, CE-T1-WIs, and T2-WIs (CT/T1/T2 matching). The GTV (7.14 cm^3^) is contoured mainly based on the boundaries of the high-density mass on CT images (A, E) and the low-intensity mass on T2-WIs (D, H), excluding the excessive exudation of contrast media (C, G). The GTV + 2 mm structure was generated by adding an isotropic 2-mm margin to the GTV for the evaluation of the dose gradient outside the GTV boundary. (C, G) The boundary of the GTV + 2 mm structure (12.33 cm^3^) almost corresponds to that of the enhancing lesion (11.66 cm^3^). CE: contrast-enhanced; CT: computed tomography; WIs: weighted images

**Figure 4:**
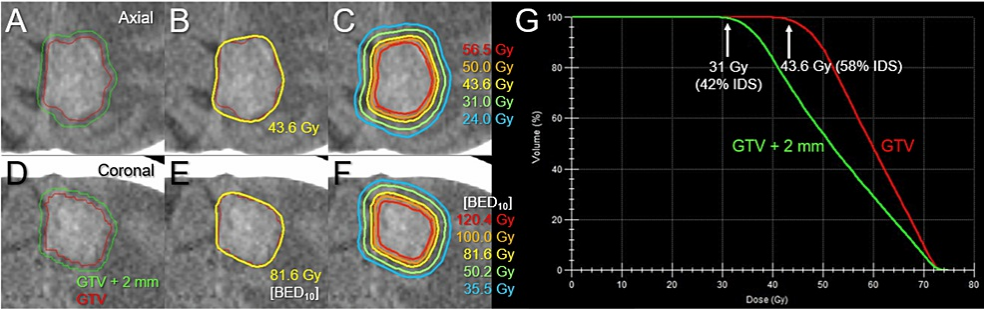
Target definition, planned dose distribution, and dose-volume histograms for the five-fraction stereotactic radiosurgery. The images show non-CE-CT images (A-F); axial images (A-C); coronal images (D-F); target contours superimposed onto CT images (A, D); representative isodose lines superimposed onto CT images (B, C, E, F); and dose-volume histogram (G). (B, E) The dose conformity of the 43.6 Gy isodose surface (IDS) to the GTV boundary, in which the corresponding biologically effective dose of 43.6 Gy is 81.6 Gy (E), based on the linear-quadratic formula with an alpha/beta ratio of 10 (BED_10_). (C) The five representative IDSs are shown as absolute doses (C) and the corresponding BED_10_ (F). (C, F, G) The concentrically-laminated steep dose increase with the BED_10_ from 81.6 Gy to 120.0 Gy inside the GTV boundary, along with the steep and moderate dose spillage outside the GTV to cover the potential microscopic tumor infiltration into the surrounding brain and other inherent uncertainties relevant to irradiation accuracy. CE: contrast-enhanced; CT: computed tomography; GTV: gross tumor volume; IDS: isodose surface (%) relative to the maximum dose (100%)

The patient had been noticing progressive dysesthesia and weakness in the right lower extremity for two months. The patient was nursing a left knee pain that developed while running, and was predominantly using the right lower extremity, and attributed the symptoms to this. Immediately before the BM diagnosis, dysesthesia in the right upper extremity and partial seizure in the right lower extremity appeared, along with difficulties in walking and writing. Steroids were administered immediately, and SRS was selected for the BM, based on multidisciplinary discussions and the patient’s preference (Figures 3, 4 and Table 1).

**Table 1:** Planning parameters for the five-fraction radiosurgery *The BED_10_s for absolute doses of 50.0 Gy, 43.6 Gy, and 31.0 Gy in 5 fractions are 100.0 Gy, 81.6 Gy, and 50.2 Gy, respectively. **The X Gy volume (vol.) is the irradiated isodose volume receiving at least X Gy, including the GTV. ***V_28.8 Gy_ is the irradiated isodose volume receiving at least 28.8 Gy outside the GTV. The actual volume includes part of the falx and the cerebrospinal fluid space. GTV: gross tumor volume; D_max_: maximum dose; BED_10_: a biologically effective dose based on the linear-quadratic formula with an alpha/beta ratio of 10; D_X%_: a minimum dose encompassing at least X% of the target volume; D_min_: minimum dose; GTV - 2 mm: GTV minus isotropic 2 mm; GTV + X mm: GTV plus an isotropic X mm margin

Structures	Volumes	Parameters	Dosimetric goals	Results
GTV	7.14 cm^3^	D_max_ (BED_10_)	≥56.4 Gy (120 Gy)	74.7 Gy (186.3 Gy)
		50.0 Gy coverage*	-	87.5%
		D_98%_ (BED_10_)	≥43.6 Gy (81.6 Gy)	45.0 Gy (85.5 Gy)
		43.6 Gy coverage*	≥98%	98.7%
		D_99.9%_ (BED_10_)	-	40.0 Gy (72.0 Gy)
		D_min_	-	36.5 Gy (63.2 Gy)
GTV – 2 mm	3.89 cm^3^	D_98%_ (BED_10_)	≥50 Gy (100 Gy)	56.3 Gy (119.7 Gy)
GTV + 1 mm	9.59 cm^3^	D_95%_ (BED_10_)	-	41.4 Gy (75.7 Gy)
		D_98%_ (BED_10_)	-	39.0 Gy (69.4 Gy)
GTV + 2 mm	12.33 cm^3^	D_95%_ (BED_10_)	-	35.6 Gy (61.0 Gy)
		D_98%_ (BED_10_)	≥31 Gy (50 Gy)	33.5 Gy (56.0 Gy)
		31.0 Gy coverage*	≥95%	99.6%
Body	-	43.6 Gy vol.**	Close to 7.14 cm^3^	8.90 cm^3^
		24.0 Gy vol.**	<20 cm^3^	20.98 cm^3^
		V_28.8 Gy_***	<7 cm^3^	9.45 cm^3^

Five-fraction SRS was initiated on the fifth day after BM diagnosis and continued for five consecutive days. Although the enhancing lesion volume was 11.66 cm^3^, the gross tumor volume (GTV) of 7.14 cm^3^ was defined based mainly on the high-density mass on computed tomography (CT) images and the low-intensity mass visible on T2-weighted images (WIs), excluding the exudation of contrast media into the surrounding brain, under multi-image co-registration and fusion (CT/T1/T2 matching) (Figure 3) [12]. The dedicated software MIM Maestro^TM^ (MIM Software, Cleveland, OH) was used for the image co-registration, fusion, and contouring. The SRS scheme included sufficient GTV coverage with a BED equivalent to a single fraction of 24 Gy, a concentrically laminated, steep dose increase inside the GTV boundary without any dose constraint to the internal dose, and an appropriate dose gradient outside the GTV [13]. The SRS was implemented using a 5-mm leaf-width multileaf collimator Agility® (Elekta AB, Stockholm, Sweden) mounted in a linac Infinity® (Elekta AB) with a 6 megavoltage (MV) X-ray flattening filter-free beam [12,13]. The patient’s head was immobilized with a thermoplastic mask [14]. The planning system employed was Monaco® (Elekta AB), and the dose calculation algorithm was an X-ray voxel Monte Carlo. The arc arrangement consisted of one coplanar arc and two non-coplanar arcs with each arc length of 180º, which are allocated at 60º intervals to divide the cranial hemisphere evenly. The collimator angles for each arc were separately set to be 90º, 45º, and 135º. The dose distribution was optimized with volumetric-modulated arcs (VMA): 98.7% of the GTV and 99.6% of the 2 mm outside the GTV were covered with 43.6 Gy (58% isodose) and 31 Gy (42% isodose), respectively, in which the BEDs for 43.6 Gy and 31 Gy are 81.6 Gy and 50.2 Gy and correspond to single doses of 24 Gy and 18 Gy, respectively, where the BED was based on the linear-quadratic model with an alpha/beta ratio of 10 (BED_10_) (Figure 4 and Table 1) [15]. Each delivery was performed under image guidance based on an XVI cone-beam CT (Elekta AB), and a HexaPOD six degrees of freedom robotic couch (Elekta AB). T2-WIs obtained after the third fraction showed no obvious changes in the configuration or location of the mass lesion [16].

**Figure 5:**
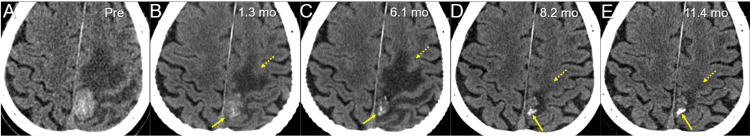
Computed tomography images before and after the five-fraction stereotactic radiosurgery The images show axial non-CE-CT images (A-E); before SRS (A); at 1.3 months (mo) after the initiation of SRS (B); at 6.1 months (C); at 8.2 months (D); and at 11.4 months (E). (A-E) These images are shown at the same magnification and coordinates under co-registration and fusions. (B) At 1.3 months, the high-density solid mass lesion (arrow in B) regressed, and its density decreased overall, although some high-density areas remained. The perilesional edema decreased (dashed arrow in B). (C) At 6.1 months, the solid mass further shrank, and its density further decreased overall, however, the high-density regions became further noticeable. In addition, the perilesional edema was significantly aggravated with the lower density than before SRS (dashed arrow in C). (D, E) At 8.2 and 11.4 months, the mass lesion remained regressed, and in part of the high-density regions, the density became even higher, suggesting calcification. The perilesional edema was markedly reduced and its extent was minimal (dashed arrows in D, E). However, the mass effect of the lesion remained, albeit moderate. CE: contrast-enhanced; CT: computed tomography; SRS: stereotactic radiosurgery

**Figure 6:**
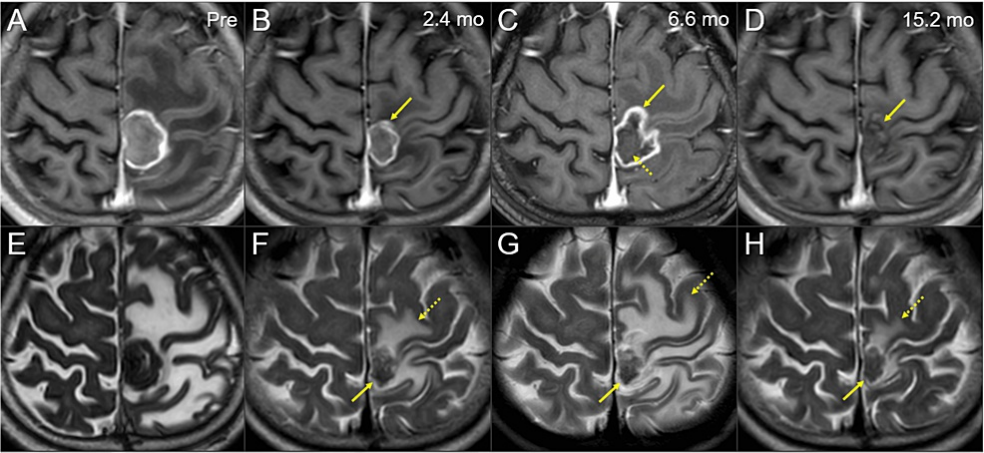
Magnetic resonance images before and after the five-fraction stereotactic radiosurgery The images show axial CE-T1-WIs (A-D); axial T2-WIs (E-H); before SRS (A, E); 2.4 months (mo) after the initiation of SRS (B, F); at 6.6 months (C, G); and at 15.2 months (D, H). (A-H) These images are shown at the same magnification and coordinates under co-registration and fusions. (B, F) The enhancing lesion (arrow in B) shrank with a decrease in the enhancement. The corresponding T2-mass (arrow in F) shrank, its intensity generally increased and became more heterogeneous, and its configuration became more irregular. The perilesional edema (dashed arrow in F) decreased remarkably. (C, G) The enhancing lesion (arrow in C) with peripheral dominance expanded remarkably and was irregular in shape, avoiding the cortex and extending into the white matter. Meanwhile, the T2-mass (arrow in G) remained regressed and its shape and intensity were the same. However, the perilesional edema (dashed arrow in G) expanded to the same degree as before SRS. Upon closer inspection, the mass lesion (dashed arrow in C) corresponding to the T2-mass was visible inside the enhancing lesion. (D, H) The lesion (arrow in D) became almost non-enhanced, and only a slightly low-intensity mass corresponding to the T2 mass was visualized. The T2-mass (arrow in H) maintained its size, and the perilesional edema (dashed arrow in H) was remarkably reduced and its degree was minimal. The images at 12.3 months (not shown) after the SRS initiation (5.2 months after BEV administration) already showed the same degree of improvement as at 15.2 months. (G, H) No apparent signal changes were observed in the contra-lateral para-central sulcus gyri. CE: contrast-enhanced; WIs: weighted images; SRS: stereotactic radiosurgery; BEV: bevacizumab

From the steroid administration and during SRS, the right-sided hemiparesis improved to the point where the patient was able to walk on his own at the completion of SRS. From 1.3 to 2.4 months after SRS, tumor shrinkage and attenuation of the perilesional edema were observed on imaging, and steroid administration was discontinued (Figures 5A, 6B, 6F).

However, 5.7 months after SRS (30.7 months after surgery), small lung oligo-metastases and new peritoneal dissemination were observed, although asymptomatic. In addition, 6.1 months after SRS, the patient noticed re-deterioration in the movement of his right lower extremity, although milder than that at the time of BM diagnosis. Images showed expansion of the enhancing lesion and perilesional edema, without enlargement of the regressed mass on CT/T2-WIs (CT/T1/T2 mismatch) (Figures 5C, 6C, 6G). The local progression was deemed to have dominance of adverse radiation effects (AREs). Therefore, in addition to the re-administration of steroids, chemotherapy was changed from irinotecan (CPT-11) to oxaliplatin (L-OHP) (Figure 1). Additionally, BEV was co-administered one month later, which resulted in remarkable improvement of the neurological symptoms, only leaving some clumsiness when starting to walk and dysesthesia in the sole of the right foot. At 1.1 months after adding BEV, the brain edema improved markedly (Figure 5D), and steroids could be discontinued. From 11.4 to 15.2 months after SRS (from 4.3 to 8.1 months after adding BEV), the enhancing effects almost disappeared, and the brain edema remained attenuated and became minimal (Figures 5E, 6D, 6H).

However, 12.6 months after SRS, the lung metastases and peritoneal dissemination progressed, and lung metastases became miliary in both lungs. Therefore, BEV was continued and a third line of chemotherapy was administered, which resulted in the progression of the extracranial metastases. At 17.3 months after SRS, a change to regorafenib was proposed but systemic therapy was discontinued at the patient’s request. Thereafter, there was no obvious deterioration in the right hemiparesis. One month later, however, gastrointestinal obstruction occurred due to the progression of the peritoneal dissemination and the patient died 18.8 months after SRS (43.9 months after surgery) (Figure 1). CT images obtained two weeks before death showed no obvious vertebral (via Batson/vertebral venous plexuses), liver, or other metastases (images not shown) [1].

## Discussion

In the BM, medical management, including co-administration of BEV (based on the assumption that radiation effects were dominant), was effective for local progression six months after SRS. Integrated diagnosis using conventional CT and magnetic resonance images (CT/T1/T2 matching) was adequate to determine the dominant pathology of local progression. Although the possibility of progression of microscopic tumor invasion into the brain could not be excluded due to the remarkable T1/T2 mismatch, the true progression of the BM was not evident during follow-up [10]. Given the dose and volume delivered to the surrounding brain (Table 1), increasing the number of dose fractions would be necessary to attenuate AREs. In addition, as no AREs were observed in the contralateral parenchyma, continued mass effects from the tumor may render the surrounding brain more susceptible to radiation injury. If the tumor had been reduced further, aggravation of the perilesional edema and relevant symptoms may have been minimal. In addition, the tumor environment may be similar to that of parasagittal meningioma, where perilesional edema is likely to develop or aggravate following SRS. The surrounding brain may also be susceptible to blood flow disturbance, such as venous congestion.

Some chemotherapeutic agents may exacerbate the parenchymal damage by penetrating the disrupted blood-brain barrier around the lesion. After switching from CPT-11 to L-OHP, the radiation effects improved, suggesting that CPT-11 may have exacerbated the radiation injury. Moreover, the addition of BEV was highly effective, facilitating early discontinuation of steroids and leading to minimal perilesional edema [5,17,18]. If BEV had been introduced early after SRS, it might have precluded AREs and enhanced the anti-tumor efficacy of the BM.

Considering that the maximum response of the BM was only partial, leaving a 1.4-cc solid mass, dose escalation to the GTV may also have been considered to further enhance the tumor shrinkage [19]. At our facility, since 2018, the general scheme of SRS has been to sufficiently cover the boundary of an enhancing lesion that is slightly larger than the corresponding T2 mass with a BED_10_ of ≥80 Gy [12,13,19]. However, the GTV in this case was defined mainly on the boundaries of the high-density mass on CT and the low-intensity mass on T2-WIs, which appeared to correspond to the actual macroscopic tumor mass [12]. Although the GTV remained regressed for 15.2 months after SRS, complete remission of the relevant symptoms was not achieved until the end. Given a generally steep dose increase inside the target boundary, the tumor response would be inferior when assigning a similar BED to the internal T2 mass boundary compared to prescribing an equivalent BED to the enhancing lesion. Furthermore, the need for a marginal dose of ≥25 Gy (BED_10_ 87.5 Gy) has been suggested in single-fraction SRS for CRC BM [2]. To achieve excellent local control with stereotactic ablative radiotherapy for lung and liver metastases from CRC, higher doses are necessary compared with those for other primaries such as non-small cell lung cancer [20]. Increasing tumor shrinkage and, if possible, achieving nearly complete local regression may lead to remission of the relevant symptoms and the sustainment of this status.

The present case also indicates another important caveat. Locally advanced cecal cancer can develop a BM without metastasizing to the liver, lungs, or vertebrae [1,8], and during systemic therapy, the rate of tumor growth may be somewhat suppressed and may increase in size more gradually. Therefore, the BM involving the motor cortex can enlarge to a considerable volume by the time the relevant symptoms appear. To diagnose BMs while they are still small, the head should preferably be scanned when performing a contrast-enhanced CT scan from the thorax to the pelvis.

One of the major limitations of this report was the lack of pathological verification of the brain lesion before and after SRS. Although the high density on CT and the low intensity on T2-WIs are typical findings of CRC BMs, the fact that BM from cecal adenocarcinoma had not been proven and the unknown pathology of the regressed lesion at 15.2 months after SRS greatly hinders the ability to draw definitive conclusions. Nevertheless, the present case forms the basis for further investigation to pursue and establish a more efficacious and safer SRS scheme in appropriate combination with systemic therapy for large CRC BM with eloquent location.

## Conclusions

An isolated BM without any other distant metastases can develop in patients receiving systemic therapy following non-curative surgery for locally advanced cecal adenocarcinoma. Multi-fraction SRS with a high marginal and internal BED to the GTV and sequential systemic therapy, including BEV, can be an efficacious, durable, and minimally-invasive treatment option for a large para-central sulcus BM from cecal adenocarcinoma. Early administration of BEV-containing regimen following SRS, dose escalation to the GTV (CT/T2 mass), and more than five fractions for ≥3 cm lesion may be considered to further improve the efficacy and safety.
